# The Impact of Congestive Heart Failure on Outcomes in Patients Hospitalized With Preeclampsia

**DOI:** 10.7759/cureus.56387

**Published:** 2024-03-18

**Authors:** Omar Elkattawy, Saahil Patel, Javier Montoya, Kanzah Sarfaraz, Sedra Alabed, Omar Gobji, Sherif Elkattawy, Jesus Romero, Fayez Shamoon

**Affiliations:** 1 Internal Medicine, Rutgers University New Jersey Medical School, Newark, USA; 2 Internal Medicine, New York Medical College, Valhalla, USA; 3 Cardiology, St. Joseph's University Medical Center, Paterson, USA; 4 Internal Medicine, Trinitas Regional Medical Center, Elizabeth, USA

**Keywords:** high-risk pregnancy, heart disease in pregnancy, congestive heart faiulre, preeclampsia, cardio-obstetrics

## Abstract

Introduction: The purpose of this study was to determine the prevalence of congestive heart failure (CHF) among patients admitted with preeclampsia as well as to analyze the independent association of CHF with in-hospital outcomes among women with preeclampsia.

Methods: Data were obtained from the National (Nationwide) Inpatient Sample (NIS) from January 2016 to December 2019. We assessed the independent association of CHF with outcomes in patients admitted with preeclampsia. Predictors of mortality in patients admitted with preeclampsia were also analyzed.

Results: Women with preeclampsia in the United States between 2016 and 2019 were included in our analysis. A total of 256,010 cases were isolated, comprising 1150 patients with preeclampsia and CHF (0.45%). Multivariate analysis demonstrated that CHF in patients with preeclampsia was independently associated with several outcomes, among them cardiac arrest (adjusted OR (aOR) 4.635, p=0.004), ventricular tachycardia (aOR 17.487, p<0.001), pulmonary embolism (aOR 6.987, p<0.001), and eclampsia (aOR 2.503, p=0.011). Conversely, we found CHF to be protective against postpartum hemorrhage (aOR 0.665, p=0.003). Among the predictors of mortality in preeclampsia are age (aOR 1.062, p=0.022), Asian or Pacific Islander race (aOR 4.695, p=0.001), and CHF (aOR 25.457, p<0.001).

Conclusions: In a large cohort of patients admitted with preeclampsia, we found the prevalence of CHF to be 0.45%. CHF was associated with several adverse outcomes as well as increased length of stay.

## Introduction

Congestive heart failure (CHF) in patients with preeclampsia represents a critical intersection of maternal health complications during pregnancy, demanding focused research and clinical attention. The combination of these two conditions poses significant challenges and underscores the need for comprehensive understanding and targeted interventions to improve patient outcomes; currently, there is a lack of research and reports addressing this crucial clinical intersection. Preeclampsia, a multi-system disorder characterized by the onset of hypertension and subsequent end-organ damage in pregnancy, significantly heightens the risk of adverse perinatal events, particularly when complicated by the development of CHF.

Globally, preeclampsia accounts for 2-8% of all pregnancy complications, resulting in over 50,00 maternal deaths and 500,000 fetal deaths [1]. In the United States, rates of preeclampsia have steadily been on the rise, with the burden of the disease disproportionately affecting Black and Indigenous pregnant patients [2]. Given this reality, it is imperative to scrutinize the causative factors of preeclampsia and potential comorbidities that heighten the risk of further complications or mortality to improve patient care, screenings, and outcomes. Despite remarkable advancements in perinatal care, the intricate interplay between preeclampsia and CHF remains a complex and dynamic area of investigation, requiring thorough exploration to enhance our understanding of the underlying pathophysiological mechanisms and develop effective management strategies. 

This comprehensive research study aims to delve into the epidemiological underpinnings, clinical predictors, and pathophysiological mechanisms linking preeclampsia with the onset and progression of CHF in affected patients. By meticulously evaluating a diverse patient cohort, this investigation endeavors to identify key risk factors, including demographic markers and clinical indicators, contributing to the heightened susceptibility to CHF in the context of preeclampsia. Furthermore, the study seeks to assess the impact of this intricate comorbidity on in-hospital adverse events, aiming to offer critical insights into optimizing patient management strategies and improving maternal and fetal outcomes. Through an exhaustive examination of prevalent risk factors, the research seeks to establish a comprehensive framework for risk assessment and early intervention, thereby contributing to a more nuanced approach to patient care and improved clinical outcomes.

Moreover, the investigation aims to shed light on the intricate relationship between preeclampsia and CHF, unraveling the complex interplay of cardiovascular dynamics and systemic changes that contribute to the development and progression of both conditions. It has widely been understood that preeclampsia predisposes the individual to a higher risk of developing heart failure later in life; analysis suggests that the lifetime risk is increased four-fold [3]. A retrospective cohort study of over 2.5 million pregnant individuals found a significant association between a pregnancy complicated by preeclampsia and later development of preserved ejection fraction heart failure with a median time to onset of 32 months [4]. One causal link that has been suggested is the increased circulation of pro-fibrotic markers and signals during preeclampsia that can later create the appropriate setting for heart failure development [5]. However, while the causal link between preeclampsia and subsequent heart failure predisposition has been an area of active investigation and discourse, there has primarily been a lack of discourse in the literature surrounding the connection between CHF and preeclampsia simultaneously in one pregnancy.

By elucidating the underlying factors associated with the concurrent onset of CHF in the context of preeclampsia, the findings of this study may prove effective in paving the way for the development of more effective diagnostic tools and preventive measures. Additionally, this study can expose potential therapeutic interventions that can mitigate the burden of these interconnected conditions on maternal health and perinatal well-being, ultimately contributing to a comprehensive and patient-centered approach to care. 

## Materials and methods

Data acquisition 

This is a retrospective database study of the National (Nationwide) Inpatient Sample (NIS). The NIS is part of the Healthcare Cost and Utilization Project (HCUP) set forth by the Agency for Healthcare Research and Quality. It utilizes the International Classification of Disease, 10th Edition, Clinical Modification (ICD-10-CM) codes for diagnosis and procedures. The data set was utilized to examine data of patients admitted between the years 2016 and 2019. Encounters with primary diagnosis of preeclampsia were selected using ICD-10 code O14. This cohort of patients was further divided into patients with CHF and patients without this comorbidity. Adult patients ≥ 18 years old were included. A total of 256,010 cases met the inclusion criteria, comprising 1150 patients with preeclampsia and CHF. IRB approval was not required as NIS provides de-identified information on patients.

Outcomes and variables 

Patient baseline characteristics such as age, race, and insurance status were extracted. Comorbidities, hospital complications, mortality rates, disposition status, length of stay, and total charges were also analyzed. 

The primary aim of the study was to assess whether or not there is a difference in outcomes (mortality, in-hospital complications, length of stay, total charges) between the patients with pre-eclampsia and CHF and the patients with pre-eclampsia and without CHF. We also analyzed the independent association of CHF with outcomes taking into account confounders such as race, sex, and comorbidities. 

Statistical analysis 

Categorical values were analyzed via Pearson Chi-square analysis and continuous variables were analyzed via independent Student’s t-test. Logistic regression was performed to generate odds ratios (ORs) with 95% confidence intervals (CIs) to assess predictors of mortality in women with preeclampsia. We also used logistic regression to assess the independent association of CHF with outcomes taking into account confounders such as age, race, and comorbidities. A p-value of <0.05 was considered statistically significant. All analyses were completed using IBM SPSS Statistics for Windows, Version 29.0 (Released 2022; IBM Corp., Armonk, New York, United States).

## Results

From January 2016 to December 2019, 256,010 cases of women with preeclampsia were isolated, comprising 1150 patients with CHF (0.45%). A statistical analysis of baseline characteristics is summarized in Table 1. Age was significantly associated with CHF in preeclampsia, with individuals in the CHF group being older on average than those in the non-CHF group (31±6.0 years vs 29± 6.3 years, p < 0.004). Disposition of patients was also found to be significant with more CHF patients being transferred to short-term hospitals (49 (4.3%) vs 2543 (1.0%), P<0.001). There was also an increased prevalence of patients with Medicaid as primary payer in the CHF group (602 (52.4%) vs 113,483 (44.6%), p<.001). Race was another significant demographic factor with a higher percentage of patients of Black race (75 (36.6%) vs 951 (22.8%), P<0.001) and Asian or Pacific Islander race (46 (4.8%) vs 389 (4.5%), P<0.001) found in the CHF group compared to non-CHF patients

**Table 1:** Baseline characteristics of the study population of preeclampsia patients stratified according to with and without congestive heart failure Data given as n (%) unless otherwise indicated.

Variable	Preeclampsia without Congestive Heart Failure, n (%)	Preeclampsia with Congestive Heart Failure, n (%)	P-value
Age in years at admission, mean ± SD	29 ± 6.3	31 ± 6.0	0.004 (T value 9.638)
Disposition of patient			0.001
Routine	244315 (95.9)	1024 (89.0)	-
Transfer to a short-term hospital	2543 (1.0)	49 (4.3)	
Transfer to other facilities, including Skilled Nursing Facility (SNF) and Intermediate Care Facility (ICF)	377 (0.1)	9 (0.8)	
Home health care (HHC)	5383 (2.1)	39 (3.4)	
Against medical advice (AMA)	2139 (0.8)	24 (2.1)	
Died in hospital	39 (0.0)	5 (0.4)	
Discharged/transferred to court/law enforcement	0 (0.0)	0 (0.0)	
Discharged alive, destination unknown	0 (0.0)	0 (0.0)	
Primary expected payer			0.001
Medicare	2777 (1.1)	40 (3.5)	-
Medicaid	113483 (44.6)	602 (52.4)	
Private insurance	126722 (49.8)	458 (39.9)	
Self-pay	5147 (2.0)	25 (2.2)	
No charge	189 (0.1)	2 (0.2)	
Other	6261 (2.5)	22 (1.9)	
Race			0.001
White	1407 (47.3)	142 (39.5)	-
Black	951 (22.8)	75 (36.6)	
Hispanic	971 (20.4)	74 (14.5)	
Asian or Pacific Islander	389 (4.5)	46 (4.8)	
Native American	383 (1.0)	22 (0.9)	
Other	244 (4.2)	18 (3.7)	

Univariate analysis results showing the associations between several comorbidities and CHF in preeclampsia are depicted in Table 2. Univariate analysis shows a higher burden of comorbidities in the CHF group including pulmonary complications such as chronic obstructive pulmonary disease (COPD), pulmonary hypertension, and obstructive sleep apnea. The cohort of patients with CHF also had a higher prevalence of other cardiovascular comorbidities including atrial fibrillation and coronary artery disease. Other medical conditions seen at higher rates among the CHF cohort were iron deficiency anemia, cerebrovascular disease, type 2 diabetes mellitus (T2DM), alcohol use disorder, liver disease, hypothyroidism, obesity, and end-stage renal disease (ESRD). In contrast, univariate analysis shows that the CHF group had a lesser burden of obstetric disorders such as gestational hypertension and gestational diabetes. 

**Table 2:** Prevalence of comorbidities in the study population of preeclampsia patients with and without congestive heart failure COPD: chronic obstructive pulmonary disease; T2DM: type 2 diabetes mellitus

Variable	Preeclampsia without Congestive Heart Failure, n (%)	Preeclampsia with Congestive Heart Failure, n (%)	P-value
Iron deficiency anemia	6509 (2.6)	127 (11.0)	0.001
COPD	19189 (7.5)	196 (17.0)	0.001
Coagulopathy	2029 (0.8)	14 (1.2)	0.109
Cerebrovascular disease	338 (0.1)	5 (0.4)	0.005
T2DM	7142 (2.8)	68 (5.9)	0.001
Hypertension	644 (0.3)	5 (0.4)	.220
Alcohol use disorder	348 (0.1)	7 (0.6)	0.001
Liver disease	974 (0.4)	20 (1.7)	0.001
Peripheral vascular disease	44 (<1%)	0 (0.0)	.656
Atrial fibrillation	159 (0.1)	14 (1.2)	0.001
Hypothyroidism	11761 (4.6)	69 (6.0)	.026
Coronary artery disease	141 (0.1)	33 (2.9)	0.001
Intracardiac thrombus	4 (<0.1%)	5 (0.4)	0.001
Pulmonary hypertension	372 (0.1)	125 (10.9)	0.001
Obstructive sleep apnea	1387 (0.5)	52 (4.5)	0.001
Gestational hypertension	20012 (7.9)	65 (5.7)	.006
Gestational diabetes	30080 (11.8)	99 (8.6)	0.001
Obesity	49272 (19.3)	357 (31.0)	0.001
End-stage renal disease	91 (0.0)	6 (0.5)	0.001

A summary of crude analysis of outcomes of patients with preeclampsia with and without CHF is included in Table 3. Independent samples T-test analysis showed that length of hospital stay was significantly longer in those with CHF status compared to those without (six days vs four days, p<0.001) as well as total hospitalization costs ($68,426 vs $32,589, P<0.001). Women with preeclampsia and CHF had a higher prevalence of in-hospital complications including cardiac arrest (14 (1.2%) vs 90 (<1%), P<0.001) and mortality (5 (0.4%) vs 39 (<0.1%), P<0.001). 

**Table 3:** Outcomes of the study population of preeclampsia patients with and without congestive heart failure Data given as n (%) except for length of stay and total charges HELLP: hemolysis, elevated liver enzymes, and low platelets; NSTEMI: non-ST-elevation myocardial infarction

Variable	Preeclampsia without Congestive Heart Failure, n (%)	Preeclampsia with Congestive Heart Failure, n (%)	P-value
Died during hospitalization	39 (<0.1%)	5 (0.4)	0.001
Length of stay (days)	4	6	0.001 (T value 14.163)
Total charges ($)	32,589	68,426	0.001 (T value 9.706)
Cardiac arrest	90 (<1%)	14 (1.2)	0.001
Ventricular tachycardia	70 (<1%)	22 (1.9)	0.001
Pulmonary embolism	104 (<0.1%)	9 (0.8)	0.001
Eclampsia	736 (0.3)	10 (0.9)	0.001
HELLP syndrome	10929 (4.3)	58 (5.0)	0.207
Shock after delivery	243 (0.1)	11 (1.0)	0.001
Postpartum hemorrhage	18591 (7.3)	72 (6.3)	0.178
Left heart catheterization	14 (0.0)	14 (1.2)	0.001
Cardiogenic shock	9 (<1%)	20 (1.7)	0.001
Mechanical ventilation	226 (0.1)	52 (4.5)	0.001
Vasopressor use	336 (0.1)	13 (1.1)	0.001
NSTEMI	34 (<1%)	16 (1.4)	0.001

**Figure 1:**
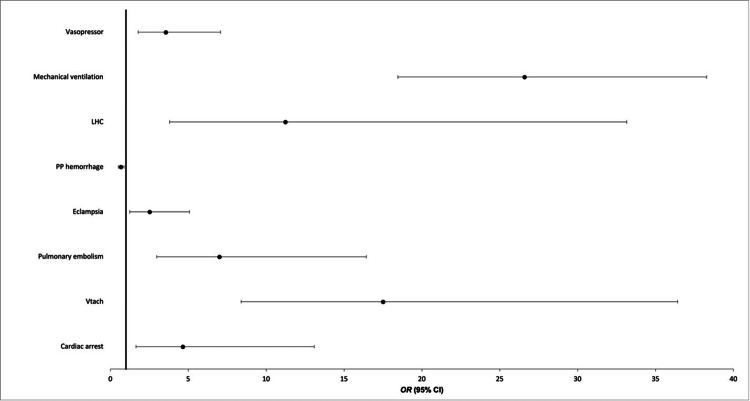
Multivariate logistic regression of outcomes of congestive heart failure in women with preeclampsia LHC: left heart catheterization; PP hemorrhage: post-partum hemorrhage

To assess the independent association of CHF with outcomes in patients with preeclampsia, a multivariate logistic regression was conducted, as summarized in Figure 1. After adjustment for confounding variables including age, race, and comorbidities, CHF was found to be independently associated with outcomes including cardiac arrest (adjusted OR (aOR) 4.635; 95%CI 1.642-13.083, p=0.004), ventricular tachycardia (aOR 17.487; 95%CI 8.396-36.421, p<0.001), pulmonary embolism (aOR 6.987; 95%CI 2.972-16.429, p<0.001), eclampsia (aOR 2.503; 95%CI 1.234-5.076, p=0.011), left heart catheterization (aOR 11.224; 95%CI 3.8-33.152; p<0.001), mechanical ventilation (aOR 26.576; 95%CI 18.451-38.278; p=<0.001), and vasopressor use (aOR 3.547; 95%CI 1.778-7.074; p<0.001). CHF was found to be protective against post-partum hemorrhage (aOR 0.665, 95%CI 0.507-0.872, P=0.003).

**Figure 2:**
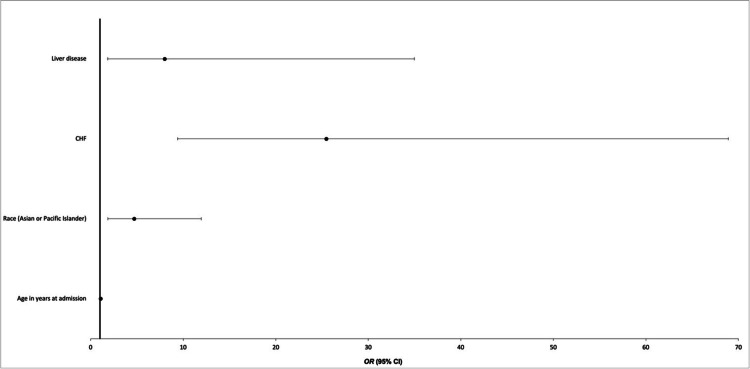
Predictors of mortality in the study population of preeclampsia patients CHF: congestive heart failure

A second multivariate logistic regression was conducted to evaluate the predictors of mortality in preeclampsia, as summarized in Figure 2. Demographic predictors that were significantly associated with an increased risk of mortality in patients with preeclampsia were age at admission (aOR 1.062; 95%CI 1.009-1.119; p=0.022) and Asian or Pacific Islander race (aOR 4.695; 95%CI 1.845-11.946; p=0.001). Comorbidities that were associated with increased risk of mortality among women with preeclampsia were CHF (aOR 25.457; 95%CI 9.405-68.901; p<0.001), and liver disease (aOR 7.985; 95%CI 1.823-34.975; P=0.006).

## Discussion

In this study, we found the prevalence of CHF in patients admitted with preeclampsia in 2016-2019 to be 0.45%. In the multivariate regression, we found CHF to be independently associated with worse outcomes including eclampsia, ventricular tachycardia, mechanical ventilation support, vasopressor use, and cardiac arrest. Conversely, we found CHF to be protective against postpartum hemorrhage. CHF was found to be a predictor of mortality among women admitted with preeclampsia, along with liver disease and demographic factors such as age and Asian or Pacific Islander race.

Our univariate analysis revealed that CHF was associated with a string of other comorbidities, among them iron deficiency anemia (IDA). This underlines the importance of comprehensive hematological monitoring in this population and the need for potential iron supplementation for hemodynamic stability [6]. Studies have revealed that in patients with CHF, the prevalence of IDA increases with the severity of heart failure. Factors contributing to the increased prevalence of IDA in CHF patients may include age, chronic inflammatory states due to heart function deterioration, and various mechanisms affecting iron metabolism [6,7]. The identification and correction of IDA have been emphasized as an essential part of comprehensive clinical management for CHF patients [8]. In addition to these findings, recent research has delved deeper into the intricacies of iron deficiency in heart failure in which a study on the nuanced relationship between iron metabolism and the pathophysiology of CHF elucidated that impaired iron homeostasis contributes significantly to the progression of heart failure [9]. This expanding body of evidence strongly supports the inclusion of routine assessment for IDA and targeted iron supplementation as a pivotal component of the holistic management of CHF patients, offering symptomatic relief and potential avenues for improving their overall cardiac function and prognosis.

Our multivariate regression showed that CHF was independently associated with many adverse outcomes, including the need for mechanical ventilation. The significant prevalence of COPD and pulmonary hypertension in patients with both CHF and preeclampsia (as our univariate analysis shows) emphasizes the intricate relationship between cardiovascular and respiratory complications during pregnancy. Among these complications that can warrant the need for mechanical ventilation is pulmonary edema. A common sequela of heart failure, pulmonary edema is a severe complication observed in preeclampsia and can lead to fatal pregnancy outcomes. The South African Saving Mother’s Report from 2014-2016 shed light on the fact that pulmonary edema accounted for 30% of all maternal deaths associated with hypertensive disorders of pregnancy [10]. These findings provide a strong indication of the need for respiratory monitoring of patients with preeclampsia due to their higher risk of developing respiratory failure and the need for mechanical ventilation. Detecting early warning signs of impaired pulmonary function could prompt treatment to stabilize the mother’s cardiopulmonary status and lead to better maternal and fetal outcomes.

Our multivariate logistic regression also shows that among other predictors of mortality, race was found to be significant. We found that Asian or Pacific Islander races are nearly five times more likely to die from preeclampsia compared to the White race. The significant results regarding patient race and morbidity/mortality outcomes indicate that more attention needs to be paid to patients of different races presenting with preeclampsia. While further validation of this finding is needed, recent research does suggest that Asian and Pacific Islander women with preeclampsia are more likely than any other racial demographic to have increased risk of cardiovascular incidents during delivery hospitalization such as arrhythmias, pulmonary edema, heart failure, and peripartum cardiomyopathy [11], which can invariably lead to increased mortality risk.

Our study has some limitations. Because our data is extracted from the NIS database, it is prone to human coding errors. In addition, patients are not followed longitudinally, therefore long-term outcomes on patients after discharge cannot be ascertained. Certain confounders such as medication use are not provided by NIS. Lastly, our analysis did not sub-stratify heart failure into reduced ejection fraction vs preserved ejection fraction. 

## Conclusions

CHF was found to be independently associated with multiple outcomes in patients with preeclampsia including mechanical ventilation use, eclampsia, and ventricular tachycardia. We also found CHF to be among the predictors of mortality in women presenting with preeclampsia, along with age and Asian and Pacific Islander races. Future research on this topic is needed to elucidate the associations between race and mortality in preeclampsia and investigate the associations between preeclampsia, CHF, and mortality. The findings of this study provide novel insights into the connection between CHF and preeclampsia in clinical settings, specifically with the associations of other comorbidities, cardiac procedures, and mortality. Thus far, this intersection has largely been undiscussed with a considerable gap in the literature addressing the unique needs and presentations of patients with concurrent CHF and preeclampsia. Further in-depth analysis is needed to fully elucidate the mechanisms behind the significant associations noted in this study.
